# The Pathological Links between Adiposity and the Carpal Tunnel Syndrome

**DOI:** 10.3390/cimb44060181

**Published:** 2022-06-08

**Authors:** Marina Ruxandra Otelea, Roxana Nartea, Florina Georgeta Popescu, Anatoli Covaleov, Brindusa Ilinca Mitoiu, Adriana Sarah Nica

**Affiliations:** 1 Clinical Department 5, Carol Davila University of Medicine and Pharmacy, 050474 Bucharest, Romania; marina.otelea@umfcd.ro; 2 Clinical Department 9, Carol Davila University of Medicine and Pharmacy, 050474 Bucharest, Romania; anatoli.covaleov@drd.umfcd.ro (A.C.); brindusa.mitoiu@umfcd.ro (B.I.M.); sarah.nica@umfcd.ro (A.S.N.); 3 National Institute for Rehabilitation, Physical Medicine and Balneoclimatology, 030079 Bucharest, Romania; 4 Department V, Internal Medicine, Victor Babeş University of Medicine and Pharmacy, 300041 Timisoara, Romania; gflorinag@yahoo.com; 5 Emergency Municipal Hospital, 300254 Timisoara, Romania

**Keywords:** obesity, carpal tunnel syndrome, metabolic syndrome, central obesity, myosteatosis

## Abstract

An association between obesity and carpal tunnel syndrome is found in many epidemiological studies. Therefore, there is a need to evaluate the physiopathological links that could explain the association between these two entities. Ectopic adipose tissue is responsible for metabolic syndrome and inflammation, and is a major risk factor for diabetes and cardiovascular diseases. Taking these elements into consideration, we conducted an extensive literature revision of the subject, considering as ectopic fat-related mechanisms the following: (a) the direct compression and the association with the metabolic syndrome of the fat deposition around the wrist, (b) the insulin resistance, dyslipidemia, inflammatory, and oxidative mechanisms related to the central deposition of the fat, (c) the impaired muscle contraction and metabolism related to myosteatosis. Each section presents the cellular pathways which are modified by the ectopic deposition of the adipose tissue and the impact in the pathogeny of the carpal tunnel syndrome. In conclusion, the experimental and clinical data support the epidemiological findings. Efforts to reduce the obesity epidemics will improve not only cardio-metabolic health but will reduce the burden of the disability-free life expectancy due to the carpal tunnel syndrome.

## 1. Introduction

Carpal tunnel syndrome (CTS) is one of the most frequent nerve entrapment syndromes, affecting 2.7/1000 of the general population [1]. It is caused by the compression of the median nerve at the wrist level. Both sensory and motor fibers are affected. Clinically, it manifests with hand pain, paresthesias of the palmar aspect of the thumb, index and middle fingers, and radial half of the ring finger and weakness of the thumb abduction and opposition with a reduction in the grip strength and in the overall hand function [2,3]. The acute form is associated with trauma, coagulopathies, and infections linked to burns [4]. The chronic form, which is the more frequent one, is classified as idiopathic and secondary [5]. The idiopathic form is more frequent in women between 40 and 60 years old [6]. According to Chammas et al., the secondary CTS is the result of the reduction of the carpal tunnel area due to abnormalities of the container (shape and positions of the bones, joint abnormalities such as arthritis, synovitis or acromegaly), of the content (inflammatory or metabolic disorders, abnormal fluid distribution, abnormal supernumerary muscle, tumors located inside the tunnel), or the variation of pressure inside the channel during repetitive movements [7]. Obesity is mentioned as a contributor to the content enlargement.

Using as search key words “carpal tunnel syndrome” AND “obesity OR waist circumference OR metabolic syndrome” we analyzed 209 human studies from the PubMed Data base, written in English. After the exclusion of reviews, case presentations and articles that showed that they were not specifically dedicated to CTS, 76 abstracts were screened, and the most relevant articles covering the epidemiology of this association were synthetized in Table S1 (see Supplementary Materials). The other articles were used to find arguments for the biological grounds of the association. After evaluating the articles and mentioning the references to experimental studies, those articles were also revised.

As any epidemiological research has to be substantiated by the identification of the biological mechanisms, this review aims to describe the current knowledge about the pathological links between obesity and CTS.

The discovery of the links between obesity and CTS is not only of theoretical interest. The ectopic fat tissue was proven to be a flexible deposit; interventions, such as physical activity and diet, have a significant benefit and, if properly managed, could reduce the burden of the CTS [5].

## 2. General Considerations about the Pathophysiology of the Carpal Tunnel Syndrome

The carpal tunnel has approximately 18–20 mm in width, 8–9 mm in depth, and 12–13 mm in length [8]. Anthropometric measurement showed that the width of the CT is correlated to the width of the palm; females have smaller CT, but the proportion of the CT components is also reduced and the ratio between the volume and the inner components was similar in both genders in flexion and extension [9].

As mentioned above, the CTS is classified as idiopathic and secondary. The CTS results from a combination of mechanical (compression and elongation of the nerve) and vascular factors (weakening the microcirculation and edema) [10]. Concerning the mechanical factors, the repetitive movements in extreme wrist flexion, particularly in conjunction with high force and non-neutral forearm pronation-supination are the key factors to increase the carpal tunnel pressure [11]. They are less important in the idiopathic form, which has most frequently a bilateral clinical expression but is the most important etiological factor in the occupational-related CTS. The vascular factors refer to the modifications in the microcirculation independent of the mechanical compression, such as the capillary fenestration and permeability or the impairment of the endothelial function encountered in pregnancy-associated CTS, metabolic syndrome (MetS), smoking, arterial-venous fistula, or hypothyroidism.

A different approach to the etiological factors is the theory of the double crush syndrome. The double crush syndrome refers to the compression of the median nerve in two different sites: proximal, at the cervical spine, and distal, at the wrist, or the coexistence of a systemic disease (diabetes, hypothyroidism) with a local compression [12]. In either a cervical lesion or a metabolic disturbance, the primary insult of the axons predisposes the peripheral nerve to more rapid deterioration during, for example, repetitive movements of the wrist. In a specialist survey, the theory of double compression was supported only by 58% [13]. However, an expert consensus agreed that the main underlying pathological mechanisms for the double crash syndrome are the following: the impaired axonal transport at one level which increases the distal mechanosensitivity, the Na channel up regulation and K channel down regulation distal and proximal from the initial lesions, the inflammation in the dorsal root ganglia after peripheral injury and the neuroma-in-continuity formation during the regeneration of the axon [13]. It is notably in this survey that even the experts who did not support the statement that a primary nerve disorder predisposes to a secondary nerve disorder by dual compression agreed on the mechanisms of the double crush syndrome attributed to general disorders.

Indirect proof of the existence of the double crush syndrome comes from the combined therapeutic approach. Initial communication about a better outcome if cervical decompression is added to the local surgical treatment had not become a common practice, although it is advisable to search and treat all possible lesions based on a risk-benefit assessment [12,14]. As for the metabolic causes as a contributor to the double crush syndrome, a meta-analysis of the functional and symptoms improvements after surgery in diabetes and non-diabetes patients found no significant differences [15]. This result does not exclude the significance of good diabetes control in preventing neuropathy, because surgery is indicated only after a long-term evolution when the disease is severe and there is a high probability of permanent damage.

**Figure 1:**
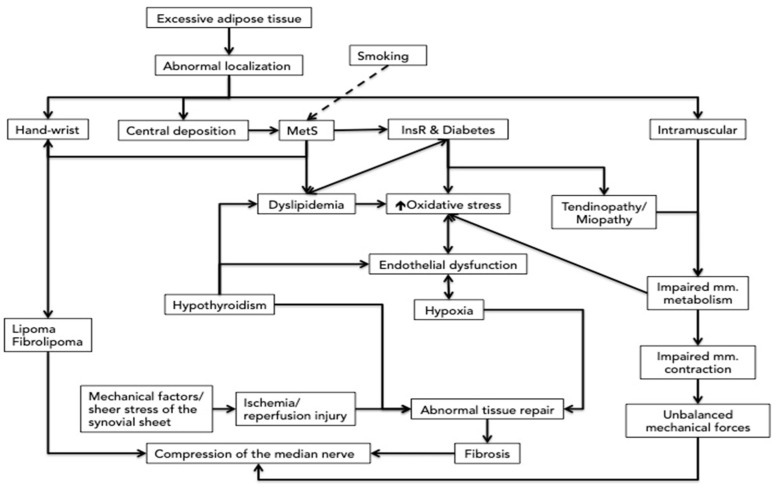
The putative links between the abnormal adipose tissue distribution and carpal tunnel syndrome. MetS = metabolic syndrome; InsR = insulin receptors.

Obesity is defined by excessive fat mass, but the white adipose tissue, the major component of the adipose tissue in adults, is far from being homogenous, and the purely quantitative definition has been complemented by terms such as “healthy” and “unhealthy” adipose tissue [16,17]. The unhealthy adipose tissue is characterized by vascular rarefaction and mitochondrial dysfunction associated with high reactive oxygen species (ROS) formation, inflammatory cells infiltration, fibrosis, and hypoxia, modifications that occur primarily in unhealthy expansion [18]. The subcutaneous fat is considered the reference for the physiological role of energy deposit of the adipose tissue; all other localizations (either visceral or muscular) are considered predominantly as an unhealthy expansion. The central deposition of obesity is the main component of metabolic syndrome (MetS) and is associated with higher cardiovascular, diabetes, cancer, and overall mortality [19,20]. The visceral adipose cells secrete IL-6 and plasminogen activator inhibitor 1, has an unbalanced adipokine profile, with higher leptin and resistin and lower adiponectin, and a different pattern of proteins [16,21,22,23,24]. Visceral fat has fewer insulin receptors and higher lipolytic activity, contributing more to dyslipidemia [24]. The pathological links between the abnormal adipose tissue and CTS are presented in Figure 1.

In muscle, lipids might accumulate inside the myocytes (intramyocellular) and between muscle fibers (extramyocellular or intermuscular) in adipocytes underneath the deep fascia of muscle [25]. The intramyocellular lipids were found both in highly trained athletes and in insulin resistance patients, therefore their significance is interpreted in context: they might represent an extra fuel to comply with the higher needs or abnormal high storage contributing to the development of muscle insulin resistance [25]. Concerning the intermuscular fat, there is more consensus about its association with insulin resistance, even if there is still debate if this abnormal coaccumulation is the cause or the consequence of insulin resistance [26,27]. A putative mechanism of intermuscular accumulation of lipids in hyperinsulinemic conditions is the increased expression of lipoprotein lipase under the insulin signal [28]. The intramuscular adipose tissue has more similarities with the visceral tissue than with the subcutaneous fat such as increased inflammatory markers [29,30]. Besides its relationship with the metabolic syndrome, the intramuscular fat also has a specific local effect on muscle contraction, which should be discussed further in this article.

## 3. The Direct Compression and the Association with the Metabolic Syndrome of the Fat Deposition around the Wrist

The deposition of adipose tissue around the wrist is common in obese persons. We can distinguish between the all-over deposition and the circumscribed one.

The all-over deposition increased the wrist circumference and was well correlated with the wrist or wrist to hip circumferences, two anthropometric indicators of MetS [31,32]. It is interesting to note that the MetS was maintained even if the wrist circumference was measured in different modes: the first study evaluated the external wrist circumference, with a tape measure positioned over the Lister tubercle of the distal radius and the distal ulna; the second one used MRI to measure the internal wrist circumference, considering the transversal profile of the wrist surrounding the bones and ligaments and excluding the adipose tissue [33]. The MRI measurement was emphasized to reflect better the anabolic effect of the insulin on the bone tissue and could be a materialization of the relation of the wrist circumference with MetS and hyperinsulinemia. Indeed, in overweight and obese persons, insulin resistance was associated with a higher level of procollagen type 1 amino propeptide, a marker of bone formation [33]. The positive relation between wrist circumference and MetS parameters was confirmed in several clinical studies, in which relation with low adiponectin/leptin ratio, HDL-cholesterol, triglycerides, and systolic blood pressure or insulin resistance was found [34,35,36]. Even more, in a longitudinal study, wrist circumference was predictive of the transition from the healthy obese phenotype to the unhealthy phenotype [37].

Lipomatous lesions are circumscribed abnormal fat-containing structures. They are less frequent than MetS. They can arise within the soft tissues, bone, neurovascular structures, and synovium [38].

Lipomatosis of the median nerve is a rare, benign condition characterized by infiltration with mature fat cells and fibrous connective tissue between nerve fascicles and the epineurium and the perineurium [39]. According to their origin and localization, Marek et al. have classified the lipomatosis lesions of the nerve as intraneural lipomas, extraneural lipomas (outside the epineurium), and lipomatosis of a nerve [40]. The last one is associated with distal nerve-territory overgrowth, affecting the soft tissue and the bony structures. The term fibro lipoma was designated for the benign tumor that develops from fibroblasts and adipocytes of the epineurium [41]. No matter the origin, inside the narrow carpal tunnel, all these benign tumors compress the nerve and lead to CTS. They should be distinct from the subcutaneous lipomas, which are benign fatty tissue tumors [42]. Lipomas are rather frequent in humans but fortunately, they rarely involve the peripheral nerves. Few cases affecting the median nerve were described in the literature, with lipomas localized at the wrist [40,41,42,43]. Direct compression by infiltration of the carpal tunnel was reported or through the local vascular flow, impairment is the putative pathological mechanism. The double crash mechanism was the explanation for the clinical benefit observed after the surgical resection of the lipoma [42,43].

## 4. Insulin Resistance, Dyslipidemia, Inflammatory and Oxidative Mechanisms Related to the Central Deposition of the Fat

Central obesity refers to the excessive abdominal fat that builds up around visceral organs and has a negative heath impact. It is the key element of MetS, the common pathological feature in diabetes and cardiovascular disease [1,44,45]. Central obesity has more important health consequences than the body mass index in general and CTS in particular [44,45]. Apparently, central androgenic obesity does not fit the female predominance of CTS [4,46]. This gender distribution was initially explained by the smaller cross-sectional area of the carpal tunnel in women and the fluid retention caused by estrogens [47,48]. From the perspective of this review, we have to underline the fact that the age range corresponds to the maximal annual incidence of CTS in both sexes between 40–60 years. Taking that into consideration we have to underline the fact that the prevalence of the Mets was found to be higher in women than in men in that age range [49,50]. Even more, a recent analysis confirmed the current trend of the increasing prevalence of MetS in women [51].

**Figure 2:**
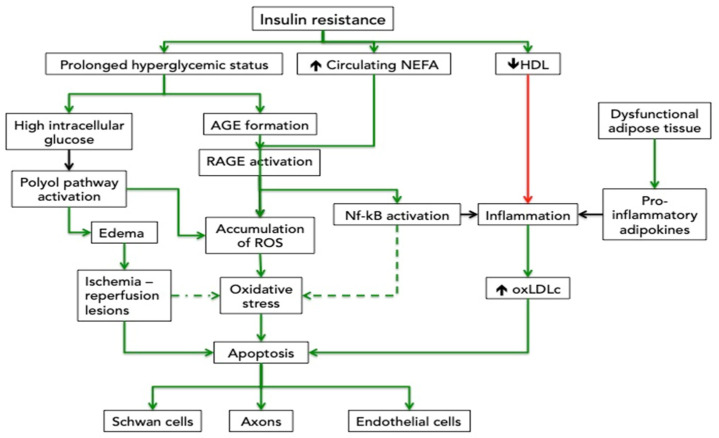
Insulin resistance and polyneuropathy. Insulin resistance is characterized by hyperglycemia, increased non-esterified fatty acid (NEFA) and dyslipidemia (low circulating HDL particles and high LDL). At cellular levels, the oxidative stress and the inflammation contribute to the destruction of Schwann cells, axons and endothelial cells. AGE= advanced glycation end products; HDL = high density lipoprotein; oxLDLc = oxidized low density lipoprotein; NEFA = non esterified fatty acids; Nf-kB = nuclear factor kappa B; RAGE = receptors for advanced glycation end products; ROS= reactive oxygen species; Green line = direct effect; red line = inhibition.

The visceral deposition of the adipose tissue has major implications on insulin resistance [23,29,30]. Related to the etiopathology of the CTS, insulin resistance directly affects the peripheral nervous system and indirectly through the vascular and muscle and tendons impairment (Figure 2) [52,53,54,55].

The excessive visceral deposition also refers to the liver. As a result, non-alcoholic fatty liver disease (NAFLD) was added to the list of diseases associated with MetS. Steatosis and insulin resistance influence each other. On one side, peripheral insulin resistance increases the lipid accumulation in hepatocytes, while on the other side, the lipid accumulation in hepatocytes contributes to the hepatic insulin resistance, aggravating the glycemic homeostasis [56] Distal symmetric neuropathy was reported in type I and type II diabetes with NAFLD [57,58]. Although encountered in diabetic patients with earlier stages of NAFLD, the peripheral polyneuropathy seems to pursue, not to precede, the onset of diabetes [58,59] In a large Korean study, the independent association between neuropathy and NAFLD in T2DM was not found or it was associated only with the last stages of fibrosis [60,61]. Hepatic insulin resistance has a role in the development of peripheral neuropathies. Further studies should clarify if there are other specific hepatic contributions besides the glucose and lipid metabolism control linking NAFLD to peripheral polyneuropathies.

### 4.1. Effects of Hyperglycemia on Nerve Impairment

The presence of MetS almost doubles the chance of peripheral neuropathy [52]. In this study, the relation between MetS and neuropathy was independent of the presence of diabetes but closely related to the waist circumference and triglyceridemia. Thus, searching for and correcting MetS components was proposed for any idiopathic neuropathy [53].

Other researchers found that peripheral neuropathy was only associated with insulin resistance and independent of MetS [54]. Indirect proof of a long subclinical evolution of neuropathy is the finding of a reduced sensory nerve action potential amplitude in the median nerve in 70% of patients at the first diagnosis of type 2 diabetes mellitus (T2DM) [55].

The neuropathy induced by hyperglycemia combines axonopathy with Schwannopathy features. The decrease in the Na+-K+ pump activity alters the axonal function [62]. The reduced trans-axonal ionic gradient and ionic currents influence neuronal transmission and conduction velocity. The Swann cell’s dysfunction is responsible for the morphological changes in the myelin sheath, the disruption of the neural support, and the impaired repairment of damaged nerves [63]. 

Several mechanisms explain these modifications. The high intracellular glucose activates other catabolic processes than glycolysis, such as the polyol pathway. The polyol pathway starts with the transformation of glucose into sorbitol, catalyzed by aldolase reductase. This reaction utilizes a hydrogen group donated by NADPH. Sorbitol is then converted into fructose via sorbitol dehydrogenase and donates a hydrogen group to NAD+, maintaining the redox balance. The second reaction does not occur in cells missing sorbitol dehydrogenase, such as the Schwann cells [64]. As consequence, these cells will acquire an unbalanced NADPH/NAD equilibrium and accumulate reactive oxygen species (ROS). These supplementary ROS are added to the already existing high level of ROS produced by the mitochondria exposed to high levels of intracellular glucose and the activation of the RAGE inside the neurons. The first organelles damaged by ROS are mitochondria. Therefore, the axons, which have a large mitochondrial pool and depend on the local energy production, are the most susceptible neuron part to the ROS effects [65]. Besides oxidative stress, sorbitol is an osmotic compound that attracts water into the cell and produces cellular edema. It has been shown that the activation of the polyol pathway induces oxidative stress, edema, and de-differentiate the Schwann cells, a process that is reversed by an inhibitor of the sorbitol formation [66]. 

### 4.2. Effects of Dyslipidemia on Nerve Impairment

There is also a significant contribution of dyslipidemia to peripheral nerves deterioration, even if the clinical association is less consistent. Some authors found an association between hypertriglyceridemia and subclinical motor and sensory axonopathy, expressed by the delay in distal latencies and decrease in conduction velocities [67,68]. Others have found this association only in those with uncontrolled glycemia or did not find any relation with triglycerides (TG) [69,70]. A systematic review concluded that the association is, however, valid in the subgroup of T2DM [71]. 

These apparently conflicting results might reflect the independent analysis of one-by-one components (e.g., TG) of the complex lipid transport system in disregard of the dynamic exchange in lipid content between the lipoprotein particles and the direct and reverse cholesterol movement. TG is an important marker of how much cholesterol is delivered to the peripheral cells but should be considered in relation to the activity of cholesteryl-ester transfer protein, which transfers TG and cholesterol esters to LDL particles, or with the activity of the lipoprotein lipase, which transforms the TG rich LDL particles into small-LDL particles [72]. The reverse transport should be taken into account as well, particularly for the low HDL-c in MetS. Experimental data show that reduction of TG and of the non-esterified fatty acid (NEFA) without normalization of glycemia is able to ameliorate the peripheral neuropathy [73]. Even more, the high level of circulating NEFA increases the superoxide production in human Schwann and endothelial cells [73]. The glycated albumin, which is more abundant in insulin resistance states, facilitates the NEFA penetration of the blood–nerve barrier [74].

The HDL molecule, which is generally in lower concentrations in plasma from patients with MetS, is not only the carrier for the reverse cholesterol transport, but has also anti-inflammatory and antioxidant properties [75]. In fact, systemic low grade inflammation is a characteristic of the MetS and is mainly part of the abnormal secretory pattern of the “unhealthy” adipocytes.

In the inflammatory context of MetS, the small low-density lipoprotein cholesterol (LDL-C) is more frequently oxidized [76]. Besides the well-known effects on endothelium, the oxidized LDL (oxLDL) could also link to the oxLDL receptor in neurons. During periods of high-fat diet-induced dyslipidemia, the oxLDL receptor in the dorsal root ganglia neurons of mice is activated and contributes to oxidative stress. Even more, in this experiment, the oxLDL effect was additive to the one derived from the hyperglycemic status [77]. The oxidative milieu favors the formation of oxysterols from cholesterol. The oxysterols induce the caspase 8 pathway and apoptosis of the neurons [78]. 

Peripheral neurons also express liver X receptors (LXR α/β) (NR1H3 and NR1H2), a cholesterol sensor that works as a transcription factor to control the lipid metabolism [79]. Gavini et al. showed that the LXR reduces the proliferation of the Schwann cells in obesity. The effect was directly related to the downregulation of neurogenin 1 by saturated fatty acids [80]. The same group found also that activation of LXR can reduce pain in diet-induced obesity. In the presence of a powerful agonist of LXR, the lipid content of the macrophages from peripheral nerves was reduced with a consequent decline in inflammation and the slower progression of the nerve lesions [81]. All the above suggest that LXR could explain symptoms of obesity-associated CTS, and should be investigated for their therapeutically potential.

A confounder in the relation between dyslipidemia and CTS that must be taken into account in epidemiological studies is hypothyroidism, as on one side, CTS is more frequent in patients with this medical condition, and on the other side, dyslipdemia, hyperinsulinemia and large waist circumference are present even in mild hypothyroidism [82]. In some studies, the body mass index (BMI) was the link between hypothyroidism and CTS [83,84]. Even in euthyroidism, the TSH level in the upper range significantly increases the chances of MetS [85].

The thyroid hormones are essential for lipid metabolism. In hypothyroidism, there is an imbalance between cholesterol synthesis and clearance. There are at least three mechanisms by which the clearance of the LDL is reduced due to the diminished synthesis of the LDL-receptor, decreased hepatic cholesterol uptake, and biliary excretion [86]. On the other side, the oxidation of the LDL-receptor increases, favoring inflammation and oxidative stress as described above [80]. The lipoprotein lipase activity is also impaired in hypothyroidism with a reduction in the TG clearance and an increase in the circulating VLDL particles [87]. The synthesis of endogenous cholesterol is not increased but the amount of available cholesterol in the liver is maintained or is even higher because the cholesterol absorption is increased and beta-oxidation in hepatocytes is decreased; the overall effect is a higher VLDL output from the liver [88]. TSH increases the activity of the hormone-sensitive lipase and the adipocyte lipolysis, mobilizing more fatty acids in the circulation [89]. The HDL synthesis and maturation are also affected, as thyroid hormones promote the synthesis of apoA1 and the efflux of cholesterol from macrophages via the ATP-binding cassette A1 [90]. Overall, hypothyroidism increases TG, LDL, and VLDL and reduces the reverse transport and the excretion of cholesterol [91]. To the already-described effects of the dislipidemia on the peripheral nerves, hypothyroidism adds the deposition of pseudo mucinous substances on the median nerve sheath and the decrease in the cross-sectional area of the carpal tunnel [84,92]. The latest effect was reversed by thyroxin treatment.

Fat is also a deposit for neurotoxic occupational hazards, and accumulation of these toxicants might delay their excretion. One of the best characterized peripheral neurotoxins with adipose tissue tropism is n-hexane [93]. The chronic n-hexane exposure initiates the formation of lysine adducts with cytoskeletal proteins; the changes in the protein structure interfere with the insertion of newly synthesized neurofilaments into the axonal cytoskeleton and the microtubule-binding and leads to the atrophy of the nerve, sensory and motor impairment [94,95]. 

Taken together, these general neural dysfunction mechanisms could be included in the expanded understanding of the double crash syndrome, defined as the coexistence of a local compression with a systemic cause of neuropathy [96]. 

### 4.3. Vascular Effects

The median nerve is affected by ischemia, secondary to the vascular remodeling or through compression of the nerve inside an abnormal narrow tunnel. Both mechanisms might be the consequences of vascular modifications that characterize MetS.

The microcirculation dysfunction is a well-known effect of T2DM and the high prevalence of the subclinical neuropathy at diagnosis is a solid argument for the deleterious effects of the long-term preexisting insulin resistance.

Inside the endothelial cells, the insulin resistance generates an imbalance between the phosphatidylinositol-3-kinase (PI3-k) and the mitogen-activated protein kinase (MAPK) pathways which results in impaired vasodilatation, a procoagulant status, the NADPH-oxidase activation, and ROS production, smooth muscle cells proliferation, augmented response to catecholamines and endothelial dysfunction [97,98,99]. In CTS, the arteriolosclerosis of the small arteries of flexor tenosynovium was observed. The narrowing of the arteriolar lumen was due to the intimal hyperplasia related to the higher expression of matrix-metalloproteinases 2 (MMP-2) [100]. A similar vascular remodeling can be induced by insulin resistance. It has been demonstrated that diet-induced insulin resistance in rats increases MMP-2 arteriolar activity, a process that was reversed by doxycycline, a blocker of the activity site of MMP-2 [101]. 

In a hyperglycemic status, the advanced glycation end products (AGE) are formed in high amounts, the specific but not the only ligand of the advanced glycation end products receptor (RAGE). Experimental data showed that “advanced oxidation protein products”, food-derived advanced glycated end products, calgranulin, amphoterin, and amyloid-b-peptide released during metabolic or oxidative cellular stress, are also able to activate RAGE. The RAGE-induced signal activates nuclear factor-kB (Nf-KB), the early growth peptide 1, and the NADPH-oxidase and yields oxidative stress, inflammatory, and prothrombotic species in atherosclerosis-prone vessels [97]. 

Hyperglycemia also influences lipid metabolism. The irreversible glycation of the LDL-C enhances its oxidative and inflammatory potential on the vascular cells [102]. Dyslipidemia generated by the enhanced lipolysis in adipocytes and de novo lipid synthesis in the hepatocytes increases the formation of the asymmetrical dimethylarginine (ADMA). ADMA inhibits the NO-synthetase, reducing the formation of nitric oxide (NO) and the vascular homeostasis [103]. Indeed, the ADMA was elevated in plasma from patients with MetS compared to controls [104]. 

MetS and hypothyroidism also share some common pathological mechanisms referring to vascular impairments such as the abnormal NO and vascular endothelial growth factor (VEGF) production [105]. A meta-analysis showed that VEGF-A is associated with diabetes, while VEGF-B and C are associated with MetS and its components [106]. In vitro, the VEGF transcription is stimulated by TSH [107]. The members of the VEGF family have pleiotropic effects in vessels; they increase the permeability, which leads to edema and further narrowing of the CT space, stimulate the multiplication of the endothelial cells with intimal thickening, the development of collateral branches, and the neovascularisation [100,108]. The neovascularization in sub-synovial connective tissue supports the high proliferation and thickening of the tendon sheet, further contributing to the narrowing of the tunnel, which is more pronounced in diabetes CTS than in patients without T2DM [109]. The process seems to be influenced by the polymorphisms of the VEGF gene, as reflected by the more frequent neuropathy in patients with T2DM and D allele of the VEGF gene [110]. Leptin, an adipokine secreted in high levels by the visceral fat, has synergic effects with VEGF, on the capillary fenestration and permeability [111,112].

The higher incidence of CTS in smokers and the relationship with MetS needs some specific comments. The odds ratio of CTS in smokers is 4.862 (95% CI, 3.991–5.925) and many studies revealed an association between smoking and central obesity, even if there is not a unanimous agreement [113,114,115,116,117]. Both body weight variation and nicotine addiction have genetic components [118,119]. In a large sample of current, former, and never smokers, investigators using the Genome-wide Complex Trait Analysis found a positive relationship between genetic factors influencing smoking habit and BMI [120]. In this study, common genetic variants predisposing to the intensity of smoking behavior and an increased BMI were identified. The influence of gender on this association is also inconsistent, with studies in which smoking influence predominated in women and others in which it was restricted only to men [116,121]. Although both smoking and female sex are well-known risk factors for CTS the interaction of these two is neglected, as it is for other pathologies [122,123]. At least from the biological perspective, prospective studies to capture the potential synergic effect of these risk factors are needed.

In what concerns the pathological mechanism, smoking has multiple negative effects on the vascular system. Active smokers have thicker arterial walls, lower flow-mediated dilatation, and response to nitroglycerine [124]. Nicotine activates the sympathetic nervous system, and the production of ROS and nitrogen species increases lipolysis in white adipose tissue and contributes to insulin resistance in muscle cells [125]. Nicotine also reduces the antioxidant enzymes (superoxide dismutase) and the activity of endothelial nitric oxide synthase, altering the endothelial function [126]. Experimental data show a slow recovery after an ischemia-reperfusion injury related to smoking. [127]. In healthy non-smokers, a 30 min passive exposure to smoking decreases the rate of oxygen consumption and the reperfusion after vascular occlusion and the capillary blood flow is significantly decreased [128,129]. In experiments conducted on the extensor digitorum longus, the chronic adaptation to hypoxia related to the blockade of hemoglobin with carbon monoxide and vasoconstriction, diminished the volume of the muscle fibers II, and increased the fiber oxidative enzyme activity [130]. 

The relation between MetS, cardiovascular disease, and CTS is endorsed also by epidemiological data. Hypertensive patients aged 30–44 years from a Finnish population-based survey, had an OR of 3.4, (95% CI 1.6–7.4) for CTS; the association with cardiac arrhythmia was even stronger (OR 10.2, 95% CI 2.7–38.4) [131]. The relation between high blood pressure and CTS was maintained after adjustment to gender, BMI, and occupational risk factors, expressed in the strain index [132]. It is also of interest that CTS might predict future risk of coronary heart disease cardiac failure, atrial fibrillation, atrioventricular heart block, and pacemaker implantation [133,134]. Most probably due to the shared risk factors (dyslipidemia, impaired metabolism, and inflammation) and the effects of MetS on the microvascular structure which lead to a reduction in the endoneurial blood flow, oxygenation, and hypoxia of the median nerve.

## 5. The Impaired Muscle Contraction and Metabolism Related to Myosteatosis 

### 5.1. Effects on Muscles and Tendons 

There is a noticeably high prevalence of musculoskeletal disorders in T2DM. Depending on the criteria used to define the musculoskeletal problems, this prevalence varies between 58.15−82.6% [135,136]. The flexor tendons of the hand have the maximum sensitivity to the deleterious effects of diabetes which might explain the 14% prevalence of CTS in diabetes [137,138]. The prevalence reaches 30% if polyneuropathy is present [138]. Fat mass and fat: muscle mass ratio is positively associated with musculoskeletal pain; persons with MetS (no matter the BMI) have more symptoms than those without MetS [139].

There are several explanations for this association. Specifically for the CTS, the altered mechanics of the hand increases the pressure inside the carpal tunnel. The median nerve flow is impaired when the intra-canal pressure exceeds 20–30 mmHg, which is 6–8 higher than the normal pressure. During certain forced movements of the hand, the lumbrical muscles enter the distal segment of the tunnel, while the flexor digitorum superficialis might enter into the proximal segment [140]. The handgrip endurance and strength showed a positive correlation with BMI and with central obesity [141,142,143]. The higher handgrip strength and hand endurance allow for longer periods of maintaining flexion or extension of the hand and increases the pressure through muscle slide inside the tunnel.

Systemic modifications related to MetS also contribute to the abnormality of the CT components. In hyperglycemia, the AGE products bound to collagen alter the collagen structure and disposal of the tendons and change the extracellular matrix [144,145]. These modifications reduce the tendon stiffness and generate an earlier response to load and a delay in repair. In an experimental model, Studentsova V et al. demonstrated a decreased flexion angle of the metatarsophalangeal and an increased gliding resistance in obese mice who lost insulin sensitivity [146]. 

Cultured tenocytes from high-fat diet mice accumulate oxLDL in the extracellular matrix. It has been shown that the oxLDL initially increases the proliferation of human tendon fibroblasts and afterward decreases the tendon content in collagen, generating tendinopathy prone to tendon rupture [147]. Combined with the impaired tendon repair in obesity these alterations will favor the contraction of the CT area [148].

The skeletal muscle structure is modified in obesity; there are fewer slow fibers (type I) and more type IIb, fast, glycolytic–dependent ones, the fatty acid oxidation is reduced and there is a loss of functional mass [149,150,151]. All these structural and functional changes converge to low fatigue resistance [151]. The obesity effects on skeletal muscle have been comprehensively revised elsewhere [152]. Briefly, the reduction of the 5′-adenosine monophosphate-activated protein kinase, mediated by insulin resistance and adiponectin depletion together with the reduced activity of peroxisome proliferator-activated receptor-γ coactivator-1α (PGC-1a) and the myocyte enhancer factor 2 are the main contributors of the switch towards the fast glycolytic types of fibers.

The ability of the muscle cells to oxidize fatty acids was assessed in muscle biopsies from lean, obese, and from obese persons who lost significant weight. The expression of several genes involved in lipid oxidation (pyruvate dehydrogenase kinase 4, carnitine palmitoyltransferase I) and the activity of the PGC-1a were also determined. The results of this study showed that lipid oxidation modified by obesity was not improved by weight loss unless it was not doubled by endurance exercise training [150]. 

In MetS, the muscle loss could be related to the sex hormone-binding globulin (SHGB), the principal plasma transporter of testosterone, oestradiol, and dihydrotestosteron. Low SHGB was found in central obesity and T2DM in epidemiological and genetic research [153,154]. Low SHGB level was associated with an increase in myostatin, a member of the transforming growth factor b and the major negative regulator of postnatal skeletal muscle growth and an important inhibitor of muscle cells regeneration via the satellite cells [155]. Following the mechanical stress usually encountered in the etiology of the CTS, the deficit in myofibrils requirement could be a relevant pathological link between MetS and CTS.

The myostatin level in obese persons was also inversely correlated with adiponectin [156]. Adiponectin is an adipokine secreted mainly in the subcutaneous fat. Subjects with higher visceral fat and lower serum adiponectin have a significantly higher risk for the development of MetS [157]. The effects of adiponectin on the skeletal muscle are both structural and functional. When high levels are present, high capillary density and type I fibers and low density of fiber II muscle fibers, and insulin sensitivity are recorded [158]. Adiponectin improves the contraction ability through regulation of Ca++ handling inside the muscle fibers in studies with adiponectin knockout mice [159]. In different studies, low adiponectin was directly or indirectly the handgrip strength. In the second study, the correlation became positive only if muscle strength was divided by body weight, showing that BMI is a significant influencer of this relation [160,161]. In the attempt to harmonize these findings, some authors proposed a physiological range of adiponectin as beneficial for muscle function, with a negative impact of either the low or the high levels, but this assumption needs to be demonstrated [162].

### 5.2. Myosteatosis

Myosteatosis represents the infiltration of fat in the muscular tissue. There are two main sites of this deposition: inside the myocytes (the intramyocellular lipid, IMCL) and within the fascia surrounding skeletal muscle (lipid infiltration or the infiltrating muscle adipose tissue, IMAT) between muscle groups. Aging, corticoid treatment, leptin deficiency, underuse, and sex steroid deficiency are risk factors for myosteatosis [163]. Myosteatosis is an ectopic fat deposition and insulin resistance is a consequence [164]. Insulin resistance is correlated to the amount of lipid content inside the muscle groups. A more significant IMAT was noticed in diabetic patients with similar lean mass [165]. 

The infiltration of muscles with lipids is an effect but also a cause of insulin resistance in obesity. The high fatty acid delivery to the muscle and low fatty acid oxidation increases the myocellular diacylglycerol content, activating the theta isoform of protein kinase C, which phosphorylates the insulin receptor substrate 1 (IRS-1). This specific phosphorylation of the insulin receptor decreases the activity of phosphatidylinositol 3-kinase and the GLUT-4 transport [166]. Of notice, the uptake of glucose and glucose oxidation is reduced in myofibrils of obese individuals, before the T2DM diagnostic criteria are met [167].

Fatty acid oxidation is part of a complex reprogramming of muscle metabolism. The activity of carnitine palmitoyltransferase (CPT-1), the carrier of lipids inside the mithocondria, and other mitochondrial processes are reduced. [168]. The impaired catabolism favors the accumulation of toxic lipids intermediates and activates the caspases, promoting apoptosis. This mechanism was described in transgenic mice with high expression of lipoprotein lipase and substantiates the deficit in contractility of the skeletal muscles [169]. This could also explain the reduction in type I fibers, which tend to accumulate more IMCL than the fast-switch oxidative ones [170]. Even in cases in which the number of type I fibers was not reduced, the shortening and maximal velocity of these fibers were negatively influenced by the IMCL [171].

Satellite cells are a pool of heterogeneous stem cells to replace the deteriorated muscle cells after microtrauma or strenuous exercise [172]. In the context of the repetitive, forceful movements of the hand which predispose to the CTS, the satellite cells biology could play a role in the ectopic fat deposition, as some of them might differentiate towards preadipocytes. Hyperglycemic or ROS abundant environment, as encountered in MetS, favors this differentiation [173,174]. Suppression of the differentiation to preadipocytes is possible in vitro and represents a potential therapeutical development for myosteatosis [175,176].

The interfibrillar deposit is represented by adipocytes infiltrating between muscle fibers. In large epidemiological studies, MetS was directly associated with sarcopenia [176]. Clinical data showed that IMAT affects the force of muscle contraction, initially attributed to the change in number, length, and metabolism of the muscle fibers [164]. Further analysis revealed that muscle force also depends on spatial arrangements (the pennation angle) relative to the direction of action and that obesity increases the volume and the pennation angle [177]. While sarcopenia is mainly an attribute of older age, the modifications of the pennation angle seem to be more pronounced in younger adults [178]. A study on the rotator cuff syndrome provided arguments for the effects of an excess IMAT on the pennation angle of the contractile fibers. The unfavorable disposal of the myofibrils determined by the IMAT resulted in a reduction in force production [179]. The lumbricals 1–2 are usually unipennate, while lumbricals 3–4 are bipennate. During the finger flexion, lumbrical muscles move into the CT. The higher the angle of the flexion, the deeper the penetration of the lumbricals inside the tunnel is and the subsequent increase in pressure [180]. Even if there are no studies specifically related to the modifications of lumbricals in obesity, it is plausible, based on the data from other skeletal muscles, that this mechanism contributes to enhanced compression and accelerates the evolution of the CTS.

Another element that leads to impaired contraction is muscle stiffness. Fatty models of muscle were stiffer and generate lower specific forces independent of the lengths of the fiber. Fat is stiffness than muscle and develops resistance to muscle shortening and transverse bulging during contraction [181]. The magnitude of the effect depends on the distribution of the fat inside the muscle mass, with the dispersed distribution being more capable of reduction of the muscle force than a single fat clamp fat [181].

Myosteatosis begins early in sedentary lifestyle. An argument was provided from the analysis of biopsies collected from healthy young subjects, before and after 3 days of muscle deconditioning. Compared to normal activity, 3 days of inactivity, upregulated perilipin, the marker of the intracellular lipid drops, and also the fatty acid-binding protein 4 in muscle tissue. High expression of genes involved in adipogenesis and fibrogenesis were also noted [182]. Comparative studies in twins with different levels of physical activity showed significant differences in the IMAT area accompanying metabolic modifications of the oxidative phosphorylation and lipid metabolism [183]. There are also suggestive data about the initiation of a neurodegenerative process during a couple of days of bed rest, with high expression of a neural cell adhesion molecule in the muscle fibers and affects the neural conduction [184,185]. 

## 6. Conclusions

In this review, we have described mechanisms that link CTS and the abnormal distribution of adiposity to illustrate the biology behind the epidemiological association. Gathering these data provided a comprehensive picture of the various mechanisms initiated by the abnormal deposition and function of the adipose tissue and the CTS. Even if this association between obesity and CTS is well established, to the best of our knowledge, this is the first attempt to synthetize the literature published about the physiopathological foundation of these epidemiological results. 

Local mechanical factors, systemic inflammatory and oxidative stress, dyslipidemia, and cross-talk between muscle and adipocyte tissue contribute to nerve compression, ischemia, and degeneration. The comprehensive literature, both experimental and clinical, adds arguments about another negative effect of obesity, namely the musculoskeletal impairment and, in particular, the CTS. Efforts to reduce the obesity epidemics will improve not only cardio-metabolic health but will reduce the burden of the disability-free life expectancy due to musculoskeletal issues.

## Data Availability

Not applicable.

## Supplementary Materials

The following supporting information can be downloaded at: https://www.mdpi.com/article/10.3390/cimb44060181/s1, Table S1: Epidemiological studies investigating the relation between carpal tunnel syndrome and obesity.
