# Supplementary material for: The Pathological Links between Adiposity and the Carpal Tunnel Syndrome
Source: Curr Issues Mol Biol. 2022 Jun 8;44(6):2646–63. doi: 10.3390/cimb44060181 (PMC9221759; doi:10.3390/cimb44060181)
Supplement: Supplementary file 1 [file cimb-44-00181-s001.zip › cimb-1737529-supplementary.pdf]

Table S1 Epidemiological studies investigating the relation between carpal tunnel syndrome and obesity

No. Crt.	Study	Type	Population	Conclusion
1	Hulkkonen S(1), Shiri R, Auvinen J, Miettunen J, Karppinen J, Ryhänen J. Risk factors of hospitalization for carpal tunnel syndrome among the general working population. Scand J Work Environ Health. 2020 Jan 1; 46(1):43-49. doi: 10.5271/sjweh.3835	longitudinal	6326 of Northern Finland Birth Cohort of 1966	After adjusting for confounders, overweight/obese participants (HR 1.69, 95% CI 1.29-2.22) were at increased risk of hospitalization for CTS
2	Alhusain FA, Almohrij M, Althukeir F, Alshater A, Alghamdi B, Masuadi E, Basudan A. Prevalence of carpal tunnel syndrome symptoms among dentists working in Riyadh. Ann Saudi Med. 2019; 39(2):104-111. doi: 10.5144/0256-4947.2019.07.03.1405.	cross sectional	232 dentists from different clinical practices	Obese dentists were also more likely to complain of CTS symptoms than dentists within normal weight limits (OR 3.66; 95% CI 1.55-8.64).
3	Thiese MS, Merryweather A, Koric A, Ott U, Wood EM, Kapellusch J, Foster J(3), Garg A, Deckow-Schaefer G, Tomich S, Kendall R, Drury DL, Wertsch J, Hegmann KT; Wistah Study Team.Association between wrist ratio and carpal tunnel syndrome: Effect modification by body mass index. Muscle Nerve. 2017;56(6):1047-1053. doi: 10.1002/mus.25692.	cross sectional	1206 employees from 17 production facilities	BMI had a significant prevalence ratio of 1.07 (102-1.06) after adjustment for age, sex, diabetes, thyroid disorder and wrist ratio.
4	Meroni R, Alberti P, Boria P, Giordano S, Cavaletti G. Distal pain and carpal tunnel syndrome diagnosis among cashiers: a longitudinal study. Int Arch Occup Environ Health. 2017;90(8):741-746. doi: 10.1007/s00420-017-1237-8.	cross sectional	198 cashiers	Overweight/obesity was present in 29.41% of cases
5	Mediouni Z, Bodin J, Dale AM, Herquelot E, Carton M, Leclerc A, Fouquet N, Dumontier C, Roquelaure Y, Evanoff BA, Descatha A. Carpal tunnel syndrome and computer exposure at work in two large complementary cohorts. BMJ Open. 2015;5(9):e008156. doi: 10.1136/bmjopen-2015-008156.	2 longitudinal cohorts	1551(in one cohort) and 771 (in the second cohort) computer workers	In multivariate analysis, there was no association between BMI or the duration of computer screen exposure with CTS
6	Fan ZJ, Harris-Adamson C, Gerr F, Eisen EA, Hegmann KT, Bao S, Silverstein B, Evanoff B, Dale AM, Thiese MS, Garg A, Kapellusch J, Burt S, Merlino L, Rempel D. Associations between workplace factors and carpal tunnel syndrome: A multi-site cross sectional study. Am J Ind Med. 2015; 58(5):509-18.	cross sectional	2981 workers from production, agriculture, construction and service sectors	Obesity workers had an OR=2.29 (1.79–2.93) for CTS.

7	Goodson JT, DeBerard MS, Wheeler AJ, Colledge AL. Occupational and biopsychosocial risk factors for carpal tunnel syndrome.J Occup Environ Med. 2014; 56(9):965-72.	case control	87 cases /74 controls	Obesity was borderline significantly related to the presence of CTS.
8	Coggon D, Ntani G, Harris EC, Linaker C, Van der Star R, Cooper C, Palmer KT. Differences in risk factors for neurophysiologically confirmed carpal tunnel syndrome and illness with similar symptoms but normal median nerve function: a case-control study. BMC Musculoskelet Disord. 2013;14:240.	case control	475 cases (Neurophysiologically confirmed)/409 senzory but not neurophysiologically CTS/799 controls	In direct comparison of neurophysiologically confirmed CTS and neurophysiologically negative patients (reference), the most notable differences were for obesity (OR 2.7, 95 % CI 1.9-3.9),
9	Burt S, Deddens JA, Crombie K, Jin Y, Wurzelbacher S, Ramsey J. A prospective study of carpal tunnel syndrome: workplace and individual risk factors. Occup Environ Med. 2013; 70(8):568-74. doi: 10.1136/oemed-2012-101287.	longitudinal	347 workers with different jobs	In the multivariate analysis, obesity had a significantly higher HR= 3.19 (1.28, 7.98).
10	Harris-Adamson C, Eisen EA, Dale AM, Evanoff B, Hegmann KT, Thiese MS, Kapellusch JM, Garg A, Burt S, Bao S, Silverstein B, Gerr F, Merlino L, Rempel D. Personal and workplace psychosocial risk factors for carpal tunnel syndrome: a pooled study cohort. Occup Environ Med. 2013;70(8):529-37.	prospective	3515 participants	A BMI greater than or equal to 30 kg/m2 almost doubled the risk of CTS and, when assessed as a continuous variable, the HR increased approximately linearly with increasing BMI.
11	Raman SR, Al-Halabi B, Hamdan E, Landry MD. Prevalence and risk factors associated with self-reported carpal tunnel syndrome (CTS) among office workers in Kuwait. BMC Res Notes. 2012 Jun 13;5:289.	cross sectional	440 office workers	OR for CTS in obese patients = 3.7 (CI:1.5-9.6), value adjusted to age, sex, previous wrist injury, number of hours of computer use, umber of hours of execise per day, comorbidities
12	Shiri R, Heliövaara M, Moilanen L, Viikari J, Liira H, Viikari-Juntura E. Associations of cardiovascular risk factors, carotid intima-media thickness and manifest atherosclerotic vascular disease with carpal tunnel syndrome. BMC Musculoskelet Disord. 2011;12:80. doi: 10.1186/1471-2474-12-80.	cross sectional	6254 subjects for the general population	In the age group: 30-44 year the obesity OR= 2.4, (CI: 1.1-5.4).Value adjusted for dislipidemia and cardiovascular disease
13	Forst L, Friedman L, Shapiro D. Carpal tunnel syndrome in spine surgeons: a pilot study. Arch Environ Occup Health. 2006;61(6):259-62.	cross sectional	107 cases in orthopedic surgeons	Predictorsfor CTS were obesity (body mass index > or = 30; adjusted odds ratio [OR] = 2.04, 95% confidence interval [CI] = 1.11-3.76) and practicing surgery for > 5 years (adjusted OR = 4.24, 95% CI = 1.54-11.69).

14	Werner RA, Franzblau A, Gell N, Hartigan AG, Ebersole M, Armstrong TJ. Incidence of carpal tunnel syndrome among automobile assembly workers and assessment of risk factors. J Occup Environ Med. 2005;47(10):1044-50	longitudinal, new cases compared to the rest	189 assembly workers/20 cases in 1 year of follow up	Baseline BMI did not increased the chance for developing CTS
15	Gell N, Werner RA, Franzblau A, Ulin SS, Armstrong TJ. A longitudinal study of industrial and clerical workers: incidence of carpal tunnel syndrome and assessment of risk factors .J Occup Rehabil. 2005;15(1):47-55.	longitudinal	432 industrial and clerical workers followed over 5.4 years	BMI was significantly more prevalent in CTS pateinets (32.3% vs 27.8%, p= 0.00)
16	Roquelaure Y, Mariel J, Dano C, Fanello S, Penneau-Fontbonne D. Prevalence, incidence and risk factors of carpal tunnel syndrome in a large footwear factory. Int J Occup Med Environ Health. 2001;14(4):357-67	baseline and longitudinal	199 workers in a footwear factory	Obesity (OR = 4.4; 95%CI: 1.1-17.1) and psychological distress at baseline (OR = 4.3; 95%CI: 1.0-18.6) were strongly predictive of CTS.
17	Atcheson SG, Ward JR, Lowe W. Concurrent medical disease in work-related carpal tunnel syndrome. Arch Intern Med. 1998; 158(14):1506-12.	obsevational	109 nontraumtaic CTS cases	The presence of a medical disease or obesity OR = 3.15 (p<-0.001) for nontraumatic CTS.
18	Lam N, Thurston A. Association of obesity, gender, age and occupation with carpal tunnel syndrome. Aust N Z J Surg. 1998;68(3):190-3.	retrospective	512 cases	CTR patients are twice as likely to be overweight (body mass index [BMI] > 25) than the general population.
19	Nathan PA, Keniston RC, Myers LD, Meadows KD. Obesity as a risk factor for slowing of sensory conduction of the median nerve in industry. A cross-sectional and longitudinal study involving 429 workers. J Occup Med. 1992;34(4):379-83	longitudinal	429 industrial workers	The risk for abnormal nerve conduction averaged between 3.5-4.1 fold greater in the obese workers than in the slender workers.
20	Wieslander G, Norbäck D, Göthe CJ, Juhlin L. Carpal tunnel syndrome (CTS) and exposure to vibration, repetitive wrist movements, and heavy manual work: a case-referent study. Br J Ind Med. 1989;46(1):43-7. doi: 10.1136/oem.46.1.43.	case control	38 cases/76 controls	OR of CTS in obese patients = 3.4 (CI:1.2- 9.8), p=0.02
21	Singjam A, Charoentanyarak K, Saengsuwan J. Prevalence and predictive factors for bilateral carpal tunnel syndrome by electrodiagnosis: A retrospective study. PLoS One. 2021;16(12):e0260578. doi: 10.1371/journal.pone.0260578.	retrospective	327 cases	BMI associated with CTS bilateral not unilateral (OR=2.9 (1.0–8.3) 0.029). After adjustement for age, sex, diabetes, duration of symptoms, the association became non-significant
22	Lores-Peniche, J.A., Huchim-Lara, O., Méndez-Domínguez, N. Carpal tunnel syndrome: Epidemiological analysis of cases treated in hospital services in Mexico. Fisioterapia. 2020; 42(2), pp. 69-74	cross sectional	3459 cases	Two thirds of patients with CTS were obese.

23	Castro Ado A, Skare TL, Nassif PA, Sakuma AK, Barros WH. Sonographic diagnosis of carpal tunnel syndrome: a study in 200 hospital workers. Radiol Bras. 2015;48(5):287-291. doi:10.1590/0100-3984.2014.0069	cross sectional	200 heathcare workers	BMI significantly higher in CTS compared to controls (p=0.0002)
24	Kouyoumdjian JA, Zanetta DM, Morita MP. Evaluation of age, body mass index, and wrist index as risk factors for carpal tunnel syndrome severity. Muscle Nerve. 2002 Jan;25(1):93-7. doi: 10.1002/mus.10007. PMID: 11754190.	case control	21 0cases/320 controls	BMI significantly higher in CTS compared to controls (p<0.001)
25	Karpitskaya Y, Novak CB, Mackinnon SE. Prevalence of smoking, obesity, diabetes mellitus, and thyroid disease in patients with carpal tunnel syndrome. Ann Plast Surg. 2002;48(3):269-73. doi: 10.1097/00000637-200203000-00007.	case control	514 cases/100 controls	There were more CTS patients than control subjects who were obese (p = 0.02; odds ratio = 1.77).
26	Geoghegan JM, Clark DI, Bainbridge LC, Smith C, Hubbard R. Risk factors in carpal tunnel syndrome. J Hand Surg Br. 2004;29(4):315-20. doi: 10.1016/j.jhsb.2004.02.009.		3391 cases	Obesity-related OR for CTS = (OR=2.06) after adjustement for age, sex, comorbidities, wrist fracture
27	Bland JD. The relationship of obesity, age, and carpal tunnel syndrome: more complex than was thought? Muscle Nerve. 2005;32(4):527-32. doi: 10.1002/mus.20408.		2408 cases	Univariate and multivariate analyses demonstrated that increased BMI is a significant independent risk factor for CTS in patients under the age of 63 years, but is less important in older patients.
28	Razavi AS, Karimi N, Bashiri F. The relationship of serum lipid profiles and obesity with the severity of carpal tunnel syndrome. Pan Afr Med J. 2021;39:90. doi: 10.11604/pamj.2021.39.90.27234.	cross sectional	118 cases	There was no significant association between obesity and the severity of CTS (p=0.054).
29	Iftikhar S, Javed MA, Kasuri MN. Frequency of Metabolic Syndrome and Its Components in Patients with Carpal Tunnel Syndrome. J Coll Physicians Surg Pak. 2016;26(5):380-3.	cross sectional	75 cases	Metabolic syndrome was found in 54 (72%) patients, out of which 54 (72%) had elevated waist circumference.
